# Molecular mechanism of modified Huanglian Wendan decoction in the treatment of polycystic ovary syndrome

**DOI:** 10.1097/MD.0000000000033212

**Published:** 2023-04-14

**Authors:** Zhaojing Wu, Tiantian Yang, Hongbo Ma

**Affiliations:** a Shandong Provincial Hospital Affiliated to Shandong First Medical University, Jinan, Shandong, China; b First College of Clinical Medicine, Shandong University of Traditional Chinese, Medicine, Jinan, Shandong, China.

**Keywords:** mechanism, modified Huanglian Wendan Decoction, molecular docking, network pharmacology, polycystic ovary syndrome

## Abstract

To investigate the mechanism of modified Huanglian Wendan decoction in the intervention of polycystic ovary syndrome (PCOS) by network pharmacology and molecular docking. The ingredients and targets of modified Huanglian Wendan decoction were retrieved from the traditional Chinese medicine Systems Pharmacology database. Related targets of PCOS were screened by Comparative Toxicogenomics Database database. Cytoscape 3.7.2 (https://cytoscape.org/) was used to draw the target network diagram of “traditional Chinese medicine - ingredient - PCOS,” STRING database was used to construct the target protein interaction network. NCA tool of Cystoscape 3.7.2 was used to carried out topology analysis on PPI network, core components and key targets were obtained. Gene ontology and Kyoto encyclopedia of genes and genomes enrichment analysis were carried out for the intersection targets by David database. AutoDockTools 1.5.6 software (https://autodock.scripps.edu/) was used to conduct molecular docking verification of key components and key targets. Ninety-one ingredients of the modified Huanglian Wendan decoction and 23,075 diseases targets were obtained, 155 Intersection targets of the drug and disease were obtained by R language, Veen plot was drawn. Gene ontology enrichment analysis obtained 432 biological processes, 67 cell components, 106 molecular functions. Fifty-four Kyoto encyclopedia of genes and genomes enrichment pathways (*P* < .05) including tumor necrosis factor, hypoxia-induced factors-1, calcium, and drug metabolism-cytochrome P450 signaling pathway. Molecular docking showed quercetin, luteolin, kaempferol, and baicalein were stable in docking with core targets. Network pharmacology and molecular docking were used to preliminarily study the mechanism of action of modified Huanglian Wendan decoction in the treatment of PCOS, which laid foundation for future experimental research and clinical application.

## 1. Introduction

Polycystic ovary syndrome (PCOS) is the most common gynecological endocrine disease in women of reproductive age, characterized by reproductive disorders, endocrine abnormalities and metabolic disorders.^[1]^ Its clinical manifestations are mainly irregular menstruation and infertility, the incidence of miscarriage, hirsutism, obesity and acne is as high as 5% to 10% in China, and 50% to 70% in infertile patients.^[2]^ At present, the pathogenesis of PCOS is complex and the exact etiology is unclear, involving many aspects such as genetic, environmental and psychological factors.

PCOS belongs to traditional Chinese medicine menstruation delay, amenorrhea or hypomenorrhea. Huanglian Wendan Decoction is from “Liuyin Tiaobian of Heat Stroke,” according to modern pharmacology that has significant lipid-lowering, hypoglycemic, anti-inflammatory and other pharmacological effects.^[3]^ Tutor in the clinical flexible use of different diseases with the concept of treatment, ingenious modified, cuscutae semen, cyperi rhizoma, poria cocos, pericarpium citri reticulatae, coptidis rhizoma, angelicae sinensis radix, persicae semen, aurantii fructus immaturus, and pinellia ternata, follow the principle of compatibility, the treatment of PCOS has a good effect, but its specific mechanism of action needs further study.

**Figure 1.**
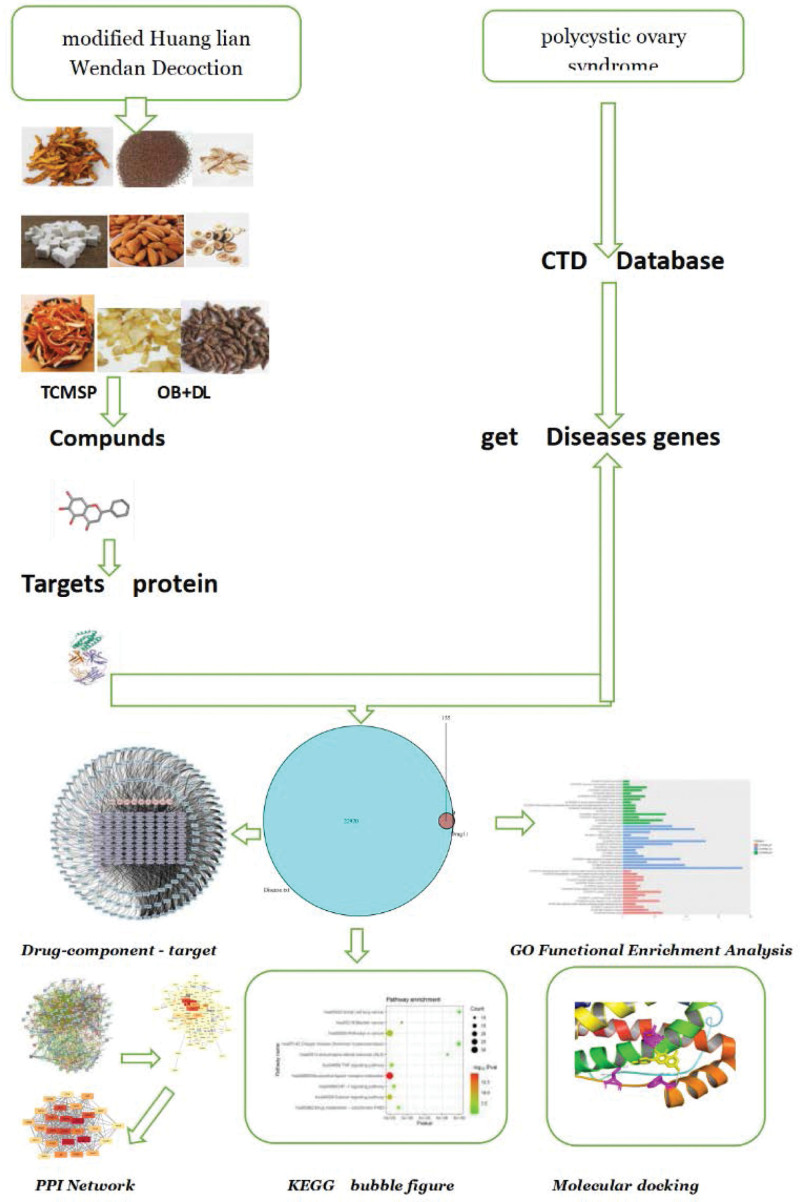
Framework based on an integration strategy of network pharmacology.

Network pharmacology is an emerging bioinformatics approach in recent years to study target molecules. Through biological function and biological active compounds of traditional Chinese medicine (TCM), combined with its mechanism of action in treating diseases, to generate the complex interaction of network. The manuscript adopted the research methods of network pharmacology, and molecular docking, analysis of modified Huanglian Wendan Decoction potential targets for the treatment of PCOS and mechanism of action, for further research on modified Huanglian Wendan Decoction treatment of PCOS provides theoretical basis (Fig. 1).

## 2. Material and methods

### 2.1. Screening active ingredients and predicting compounds targets of modified Huanglian Wendan decoction

The ingredients of cuscutae semen, cyperi rhizoma, poria cocos, citrus reticulata, coptidis rhizoma, angelicae sinensis radix, persicae semen, aurantii fructus immaturus, and arum ternatum thunb were gathered from TCMSP^[4]^ (https://tcmsp-e.com/tcmsp.php) based on the parameters related to drug metabolism kinetics; oral bioavailability ≥ 30% and drug similarity ≥ 0.18 were the selecting conditions.

### 2.2. Collecting targets of PCOS

Enter the keyword “polycystic ovary syndrome” into the the comparative toxicogenomics database (CTD) database^[5]^ (http://ctdbase.org/) to obtain the known target information related to this disease. Uniprot^[6]^ (https://www.rcsb.org/) database was used to standardize the names of target genes.

### 2.3. Intersection target acquisition

The intersection of drugs component targets and diseases were obtained by R language and Venn diagram was drawn.

### 2.4. Ingredients-targets-TCM network

The targets of intersection between modified Huanglian Wendan decoction and PCOS were taken to establish the network diagram using Cytoscape 3.7.2,^[7]^ the topological properties of network were analyzed. To better clarify the mechanism of the treatment of PCOS with modified Huanglian Wendan Decoction, the core network was selected based on topological parameters, degree and the betweenness centrality were key indexes to estimate the importance of nodes, as the degree and betweenness centrality increase, the nodes are more important in the network.

### 2.5. Target protein interaction analysis

The target at the intersection of TCM ingredients and diseases was imported into the STRING database^[8]^ (http://string.embl.de/), the species was set as “Homo Sapiens,” the lowest interaction threshold was set as “medium confidence,” and the target protein interaction network was obtained.

### 2.6. Gene ontology (GO) and Kyoto encyclopedia of genes and genomes (KEGG) pathway enrichment analysis

DAVID (https://david.ncifcrf.gov/) was used to GO^[9]^ and KEGG pathway enrichment ^[10]^ analysis on the intersection target proteins, obtained the signal pathways of modified Huanglian Wendan decoction for the intervention of PCOS.

### 2.7. Docking verification of core target molecules

The Cytoscape3.7.2 CNA widget was used to screen the core targets and effective active compounds according to the degree value. The chemical structure of active components in MOL2 format of TCMSP was retrieved from the database, which was re-retrieved in Pubchem database and saved in SDF format^[11]^ (https://pubchem.ncbi.nlm.nih.gov/). Search the PDB database (https://www.rcsb.org/) for the protein and its crystal structure. After dehydration and hydrogenation, the protein was stored in PDB format with Autodoc 1.5.6 software (https://autodock.scripps.edu/). PyMol software^[12]^ was used to analyze the important proteins in the active ingredients and target network whose degree value was greater than the average.

## 3. Results

### 3.1. Ingredients composition and target prediction of modified Huanglian Wendan decoction

From the TCMSP databases, obtained 650 ingredients, through absorption, distribution, metabolism, and excretion (oral bioavailability ≥ 30%, drug similarity ≥ 0.18) screening (Table 1, Table S1, Supplemental Digital Content, http://links.lww.com/MD/I636 and Table S2, Supplemental Digital Content, http://links.lww.com/MD/I637). After screening again to remove duplication, 91 modified active ingredients of modified Huanglian Wendan decoction were identified. A total of 164 targets were predicted through UniProt gene annotation search using these 91 compounds, including 9 Cuscutae Semen, 10 Cyperi Rhizoma, 13 Poria Cocos, 4 pericarpium citri reticulatae, 9 Coptidis Rhizoma, 2 Angelicae Sinensis Radix, 16 Persicae Semen, 16 Aurantii Fructus Immaturus, and 10 Pinellia ternata.

**Table 1:** Compounds.

MOL number	Compound	OB%	DL	Herbs
MOL000006	Luteolin	36.16	0.25	Aurantii fructus immaturus
MOL001798	Neohesperidin_qt	71.17	0.27	Aurantii fructus immaturus
MOL001803	Sinensetin	50.56	0.45	aurantii fructus immaturus
MOL001941	Ammidin	34.55	0.22	Aurantii fructus immaturus
MOL002914	Eriodyctiol (flavanone)	41.35	0.24	Aurantii fructus immaturus
MOL004328	Naringenin	59.29	0.21	Aurantii fructus immaturus
MOL005849	Didymin	38.55	0.24	Aurantii fructus immaturus
MOL007879	Tetramethoxyluteolin	43.68	0.37	Aurantii fructus immaturus
MOL009053	4- [(2S, 3R)-5-[(E)-3-hydroxyprop-1-enyl]-7-methoxy-3-methylol-2, 3-dihydrobenzofuran-2-yl]-2-methoxy-phenol	50.76	0.39	Aurantii fructus immaturus
MOL013277	Isosinensetin	51.15	0.44	Aurantii fructus immaturus
MOL013279	5, 7, 4’-Trimethylapigenin	39.83	0.3	Aurantii fructus immaturus
MOL013430	Prangenin	43.6	0.29	Aurantii fructus immaturus
MOL013435	Poncimarin	63.62	0.35	Aurantii fructus immaturus
MOL013436	Isoponcimarin	63.28	0.31	Aurantii fructus immaturus
MOL013437	6-Methoxy aurapten	31.24	0.3	Aurantii fructus immaturus
MOL013440	Citrusin B	40.8	0.71	Aurantii fructus immaturus
MOL000359	Sitosterol	36.91	0.75	Pericarpium citri reticulatae
MOL005100	5, 7-dihydroxy-2-(3-hydroxy-4-methoxyphenyl) chroman-4-1	47.74	0.27	pericarpium citri reticulatae
MOL005815	Citromitin	86.9	0.51	Pericarpium citri reticulatae
MOL005828	Nobiletin	61.67	0.52	Pericarpium citri reticulatae
MOL003044	Chryseriol	35.85	0.27	Cyperi rhizoma
MOL003542	8-Isopentenyl-kaempferol	38.04	0.39	Cyperi rhizoma
MOL004053	Isodalbergin	35.45	0.2	Cyperi rhizoma
MOL004058	Khell	33.19	0.19	Cyperi rhizoma
MOL004059	Khellol glucoside	74.96	0.72	Cyperi rhizoma
MOL004068	Rosenonolactone	79.84	0.37	Cyperi rhizoma
MOL004071	Hyndarin	73.94	0.64	Cyperi rhizoma
MOL004074	Stigmasterol glucoside_qt	43.83	0.76	Cyperi rhizoma
MOL004077	Sugeonyl acetate	45.08	0.2	Cyperi rhizoma
MOL010489	Resivit	30.84	0.27	Cyperi rhizoma
MOL000098	Quercetin	46.43	0.28	Cuscutae semen
MOL000184	NSC63551	39.25	0.76	Cuscutae semen
MOL000354	Isorhamnetin	49.6	0.31	Cuscutae semen
MOL000422	Kaempferol	41.88	0.24	Cuscutae semen
MOL000953	CLR	37.87	0.68	Cuscutae semen
MOL001558	Sesamin	56.55	0.83	Cuscutae semen
MOL005043	Campest-5-en-3beta-ol	37.58	0.71	Cuscutae semen
MOL005440	Isofucosterol	43.78	0.76	Cuscutae semen
MOL005944	Matrine	63.77	0.25	Cuscutae semen
MOL000493	Campesterol	37.58	0.71	Persicae semen
MOL001323	Sitosterol alpha1	43.28	0.78	Persicae semen
MOL001328	2, 3-didehydro GA70	63.29	0.5	Persicae semen
MOL001329	2, 3-didehydro GA77	88.08	0.53	Persicae semen
MOL001339	GA119	76.36	0.49	Persicae semen
MOL001340	GA120	84.85	0.45	Persicae semen
MOL001342	GA121-isolactone	72.7	0.54	Persicae semen
MOL001344	GA122-isolactone	88.11	0.54	Persicae semen
MOL001349	4a-formyl-7alpha-hydroxy-1-methyl-8-methylidene-4aalpha, 4bbeta-gibbane-1alpha, 10beta-dicarboxylic acid	88.6	0.46	Persicae semen
MOL001351	Gibberellin A44	101.61	0.54	Persicae semen
MOL001352	GA54	64.21	0.53	Persicae semen
MOL001353	GA60	93.17	0.53	Persicae semen
MOL001355	GA63	65.54	0.54	Persicae semen
MOL001358	Gibberellin 7	73.8	0.5	Persicae semen
MOL001360	GA77	87.89	0.53	Persicae semen
MOL001361	GA87	68.85	0.57	Persicae semen
MOL001368	3-O-p-coumaroylquinic acid	37.63	0.29	Persicae semen
MOL000273	(2R)-2- [(3S, 5R, 10S, 13R, 14R, 16R, 17R)-3, 16-dihydroxy-4, 4, 10, 13, 14-pentamethyl-2, 3, 5, 6, 12, 15, 16, 17-octahydro-1H-cyclopenta [a] phenanthren-17-yl]-6-methylhept-5-enoic acid	30.93	0.81	Poria cocos
MOL000275	Trametenolic acid	38.71	0.8	Poria cocos
MOL000276	7, 9 (11)-dehydropachymic acid	35.11	0.81	Poria cocos
MOL000279	Cerevisterol	37.96	0.77	Poria cocos
MOL000280	(2R)-2- [(3S, 5R, 10S, 13R, 14R, 16R, 17R)-3, 16-dihydroxy-4, 4, 10, 13, 14-pentamethyl-2, 3, 5, 6, 12, 15, 16, 17-octahydro-1H-cyclopenta [a] phenanthren-17-yl]-5-isopropyl-hex-5-enoic acid	31.07	0.82	Poria cocos
MOL000282	Ergosta-7, 22E-dien-3beta-ol	43.51	0.72	Poria cocos
MOL000283	Ergosterol peroxide	40.36	0.81	Poria cocos
MOL000287	3beta-Hydroxy-24-methylene-8-lanostene-21-oic acid	38.7	0.81	Poria cocos
MOL000289	Pachymic acid	33.63	0.81	Poria cocos
MOL000290	Poricoic acid A	30.61	0.76	Poria cocos
MOL000291	Poricoic acid B	30.52	0.75	Poria cocos
MOL000292	Poricoic acid C	38.15	0.75	Poria cocos
MOL000296	Hederagenin	36.91	0.75	Poria cocos
MOL000519	Coniferin	31.11	0.32	Pinellia ternata
MOL001755	24-Ethylcholest-4-en-3-one	36.08	0.76	Pinellia ternata
MOL002670	Cavidine	35.64	0.81	Pinellia ternata
MOL002714	Baicalein	33.52	0.21	Pinellia ternata
MOL002776	Baicalin	40.12	0.75	Pinellia ternata
MOL003578	Cycloartenol	38.69	0.78	Pinellia ternata
MOL005030	Gondoic acid	30.7	0.2	Pinellia ternata
MOL006936	10,13-eicosadienoic	39.99	0.2	Pinellia ternata
MOL006937	12, 13-epoxy-9-hydroxynonadeca-7, 10-dienoic acid	42.15	0.24	Pinellia ternata
MOL006957	(3S, 6S)-3-(benzyl)-6-(4-hydroxybenzyl) piperazine-2, 5-quinone	46.89	0.27	Pinellia ternata
MOL006967	Beta-D-Ribofuranoside, xanthine-9	44.72	0.21	Pinellia ternata
MOL000358	Beta-sitosterol	36.91	0.75	Angelicae sinensis radix
MOL000449	Stigmasterol	43.83	0.76	Angelicae sinensis radix
MOL000622	Magnograndiolide	63.71	0.19	Coptidis rhizoma
MOL000785	Palmatine	64.6	0.65	Coptidis rhizoma
MOL001454	Berberine	36.86	0.78	Coptidis rhizoma
MOL002894	Berberrubine	35.74	0.73	Coptidis rhizoma
MOL002897	Epiberberine	43.09	0.78	Coptidis rhizoma
MOL002903	(R)-Canadine	55.37	0.77	Coptidis rhizoma
MOL002907	Corchoroside A_qt	104.95	0.78	Coptidis rhizoma
MOL013352	Obacunone	43.29	0.77	Coptidis rhizoma
MOL002904	Berlambine	36.68	0.82	Coptidis rhizoma

DL = drug similarity, OB = oral bioavailability.

### 3.2. Obtained the disease targets

Through CTD databases, obtained 23,075 disease targets of PCOS (Supplemental Digital Content 3, http://links.lww.com/MD/I638).

### 3.3. Screening of common targets between compound ingredients and diseases

**Figure 2.**
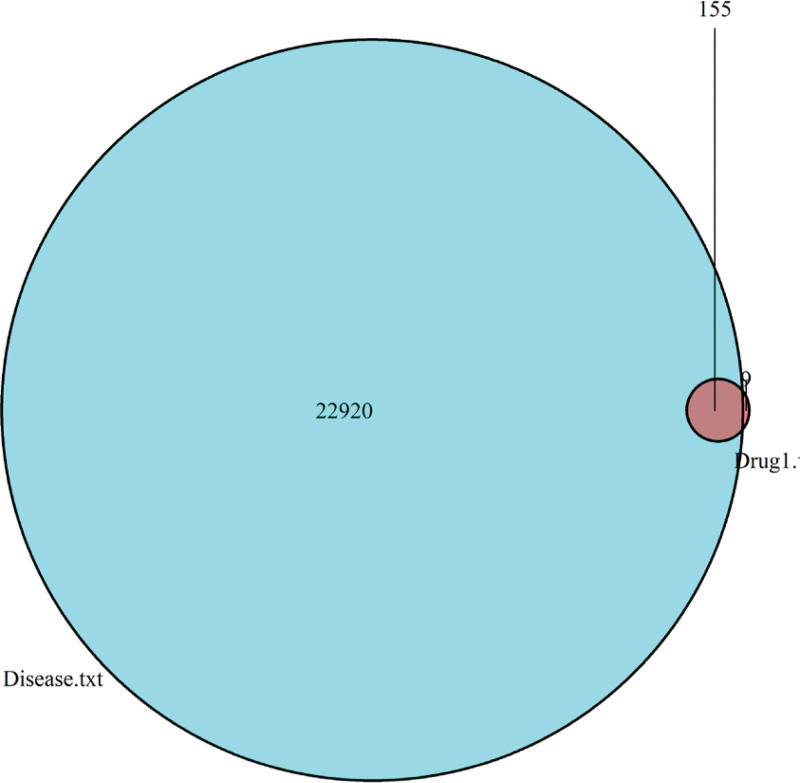
Venn diagram: screening of common targets between compound ingredients and diseases.

Data on PCOS-related targets retrieved from CTD databases were integrated, resulting in the retrieval of 23,075 targets. These targets were compared with the predicted targets, and 155 common targets were filtered as the key targets for testing the anti-PCOS activity of the ingredients (Fig. 2, Supplemental Digital Content 4, http://links.lww.com/MD/I639).

### 3.4. The network of ingredients-targets-TCM

**Figure 3.**
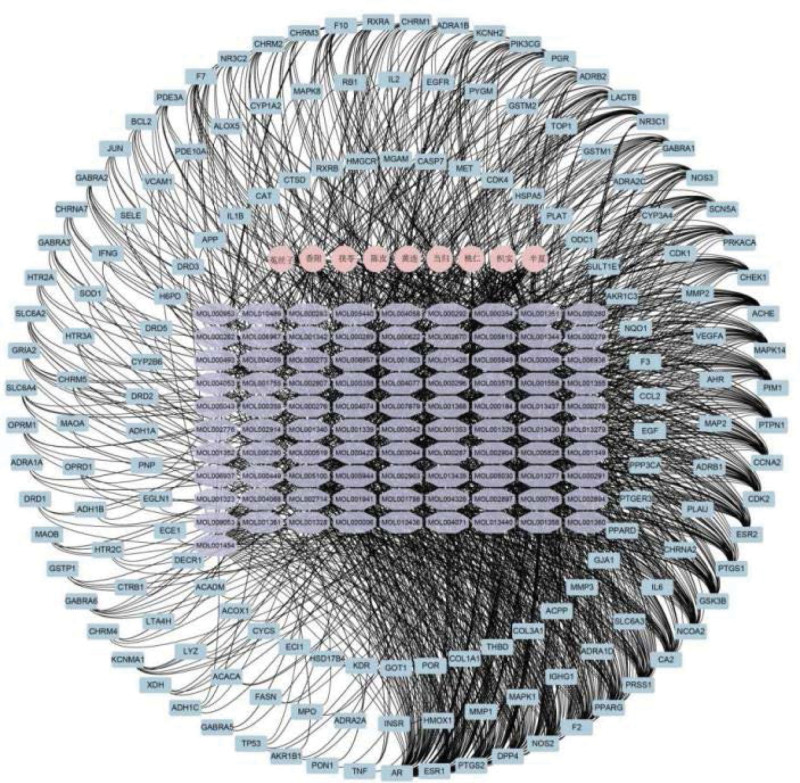
Interaction network to indicate herbs-ingredients- targets composited of, herbs is pink, ingredients is purple and targets is blue.

Cytoscape 3.7.2 constructed network of TCM, ingredients and targets (Fig. 3, Supplemental Digital Content 1, http://links.lww.com/MD/I636), consisting of 9 herbal nodes, 91 ingredients nodes, and 155 protein nodes. Among the 91 compounds, the active ingredients closely related to PCOS were quercetin, luteolin, kaempferol, baicalein, β-sitosterol, isorhamnetin, stigmasterol, and nobiletin.

### 3.5. PPI network analysis

**Figure 4.**
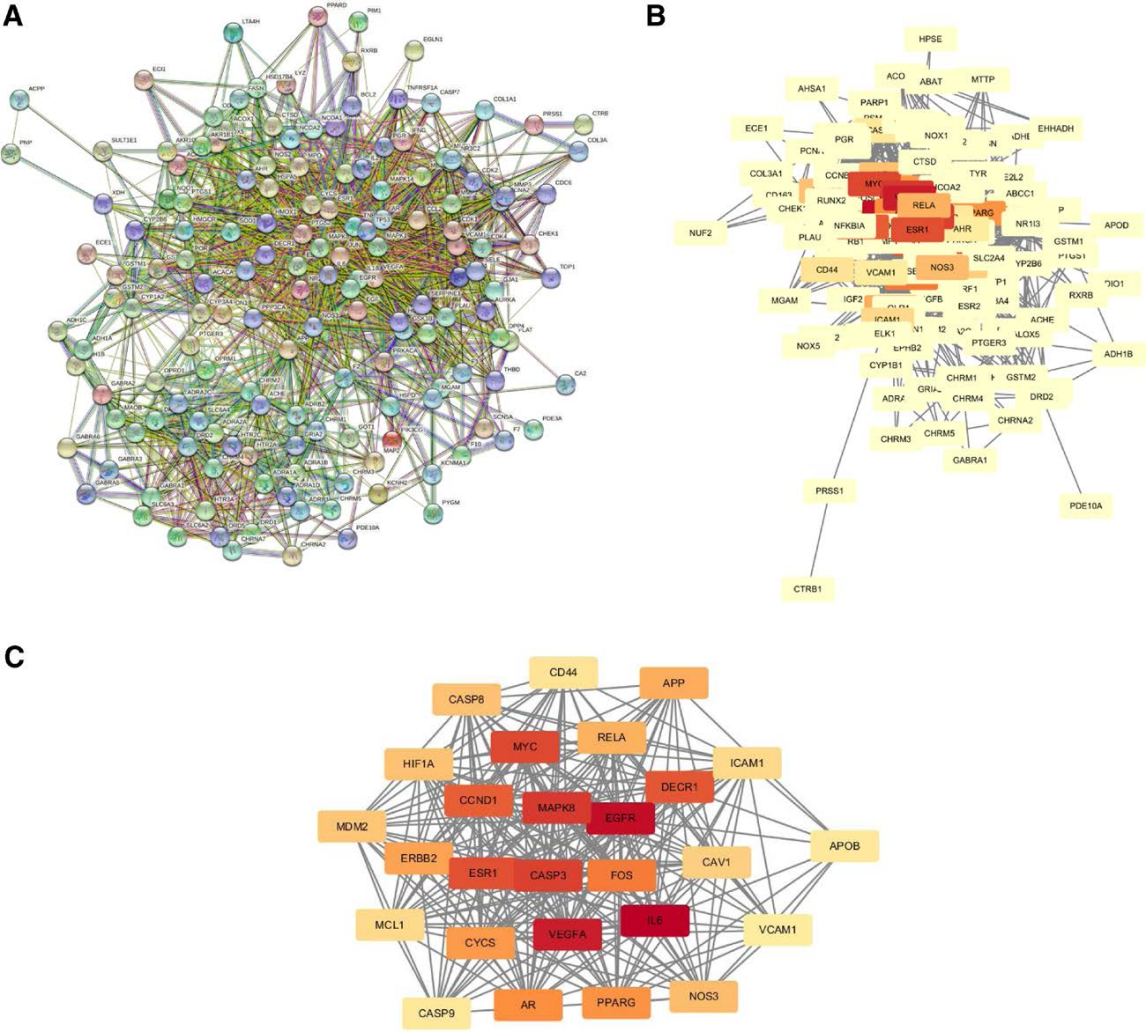
The process of topological screening for the PPI network.

Will intersect the target were input into STRING, remove the unconnected target, the PPI network was obtained, save the TSV file. Used Cytoscape 3.7.2 for further analysis, showed that the network contained 153 nodes and 1691 edges. Network analysis was carried out through Network Viz, and 27 core targets with an average degree value >36 were identified (Table 2). The network diagram of core targets and noncore targets was constructed (Fig. 4A–C). Finally, different HUB computing methods were used to calculate the top 10 genes (Table 3), and it can be concluded that interleukin-6 (IL-6), vascular endothelial growth factor A (VEGFA), epidermal growth factor receptor (EGFR), mitogen-activated protein kinase 8 (MAPK8), recombinant cyclin D1 (CCND1), caspase 3 (CASP3) and FOS are common genes after HUB algorithm, and the screened active ingredients will be molecular docked.

**Table 2:** The information of 27 core targets ranked by degree.

UniprotID	Gene symbol	Protein name
P05231	IL-6	Interleukin-6
P00533	EGFR	Epidermal growth factor receptor
P15692	VEGFA	Vascular Endothelial Growth Factor
P45983	MAPK8	Mitogen-activated protein kinase 8
P42574	CASP3	Caspase 3
P01106	MYC	Myc proto-oncogene protein
P03372	ESR1	Estrogen receptor
Q16698	DECR1	2, 4-dienoyl-CoA reductase [(3E)-enoyl-CoA-producing1]
P24385	CCND1	G1/S-specific cyclin-D1
P01100	FOS	Protein c-Fos
P10275	AR	Androgen receptor
P37231	PPARG	Peroxisome proliferator-activated receptor gamma
P99999	CYCS	Cytochrome c
P04626	ERBB-2	Receptor tyrosine-protein kinase erbB-2
P05067	APP	Amyloid-beta precursor protein
Q04206	RELA	Transcription factor p65
P29474	NOS3	Nitric oxide synthase
Q16665	HIF1A	Hypoxia-inducible factor 1 alpha
Q14790	CASP8	Caspase-8
Q00987	MDM2	E3 ubiquitin-protein ligase Mdm2
Q03135	CAV1	Caveolin-1
Q07820	MCL1	Induced myeloid leukemia cell differentiation protein Mcl 1
P05362	ICAM1	Intercellular adhesion molecule 1
P16070	CD44	CD44 antigen
P55211	CASP9	Caspase-9
P04114	APOB	Apolipoprotein B-100, Apo B-100
P19320	VCAM1	Vascular cell adhesion protein 1

CASP3 = Caspase 3, CCND1 = recombinant cyclin D1, EGFR = epidermal growth factor receptor, HIF = hypoxia-induced factors, IL-6 = interleukin-6, MAPK8 = mitogen-activated protein kinase 8, VEGFA = vascular endothelial growth factor A.

**Table 3:** The top 10 Hub genes ranked with different algorithms.

Category	Rank method in Cyto Hubba			
	Degree	MNC	EPC	Closeness
Gene symbol top 10	IL-6	IL-6	CASP3	IL-6
	EGFR	EGFR	IL-6	EGFR
	VEGFA	VEGFA	EGFR	VEGFA
	MAPK8	MAPK8	VEGFA	MAPK8
	CASP3	CASP3	MAPK8	CASP3
	MYC	MYC	MYC	TNF
	ESR1	ESR1	ESR1	DECR1
	DECR1	DECR1	CCND1	ESR1
	CCND1	CCND1	FOS	CCND1
	FOS	FOS	AR	FOS

CASP3 = Caspase 3, CCND1 = recombinant cyclin D1, EGFR = epidermal growth factor receptor, IL-6 = interleukin-6, MAPK8 = mitogen-activated protein kinase 8, TNF = tumor necrosis factor, VEGFA = vascular endothelial growth factor A.

### 3.6. GO functional enrichment analysis and KEGG pathway analysis

**Figure 5.**
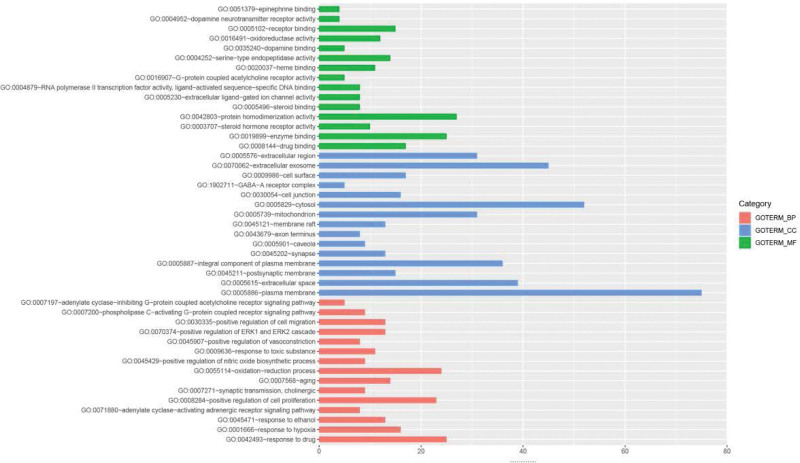
Bioanalysis of GO, include BP, MF, and CC. BP = biological processes, CC = cellular component, MF = molecular function, GO = gene ontology.

To further explore the various mechanisms of the anti-PCOS activity of modified Huanglian Wendan decoction, we performed a GO enrichment analysis, obtained 432 biological processes (BP) (Supplemental Digital Content 5, http://links.lww.com/MD/I640), 67 cellular component (CC) (Supplemental Digital Content 6, http://links.lww.com/MD/I641) 106 and molecular function (MF) (Supplemental Digital Content 7, http://links.lww.com/MD/I642) of the 155 targets. The Top 15 enriched BP, MF and CC were selected and represented by a bar chart (Fig. 5). Among them, BP involves: response to drug, response to hypoxia, adenylate cyclase-activating adrenergic receptor signaling pathway, positive regulation of cell proliferation, synaptic transmission, cholinergic, oxidation-reduction process, positive regulation of nitric oxide biosynthetic process, positive regulation of vasoconstriction, positive regulation of extracellular regulated protein kinases1 (ERK1) and extracellular regulated protein kinases2 (ERK2) cascade. CC involves: plasma membrane, extracellular space, postsynaptic membrane, integral component of plasma membrane, synapse, caveola, axon terminus, membrane raft, mitochondrion, cytosol, cell junction, cell surface, γ-aminobutyric acid (GABA)-A receptor complex, extracellular exosome. MF involves: drug binding, enzyme binding, steroid hormone receptor activity, protein homodimerization activity, RNA polymerase II transcription factor activity, ligand-activated sequence-specific DNA binding, G-protein coupled acetylcholine receptor activity, steroid binding, extracellular ligand-gated ion channel activity, oxidoreductase activity, heme binding, serine-type endopeptidase activity, dopamine binding, dopamine neurotransmitter receptor activity.

**Figure 6.**
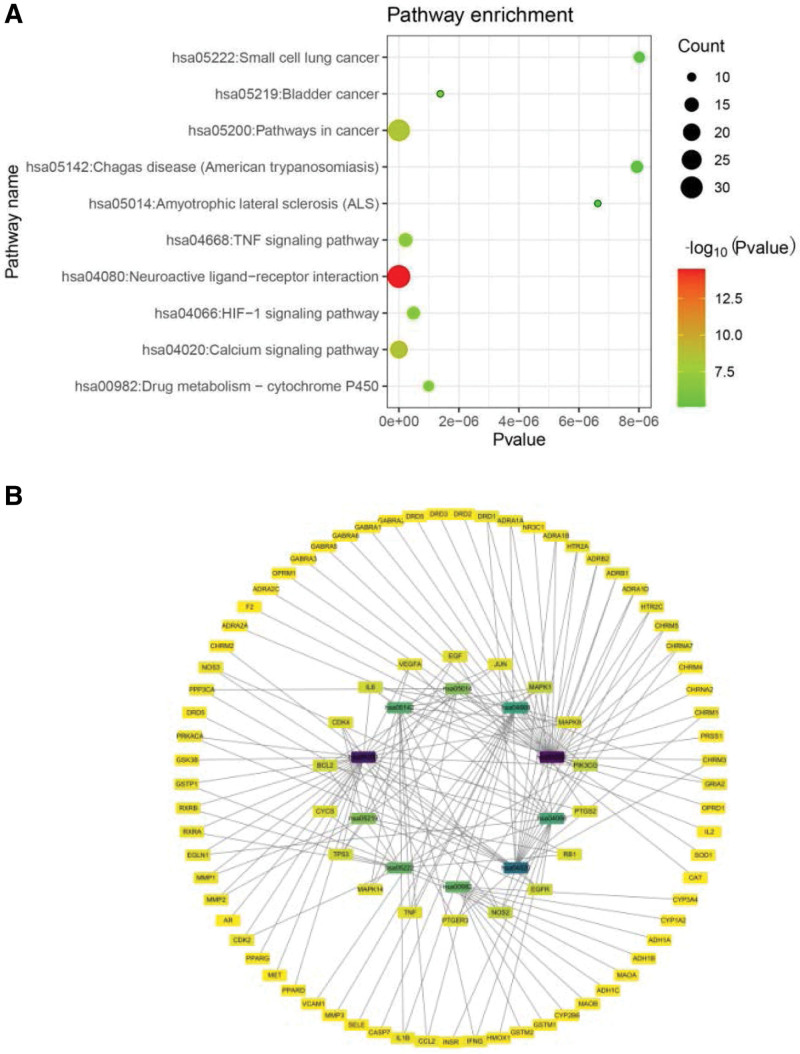
(A) KEGG bubble figure. (B) Pathway and target gene figure. (The darker nodes represent paths, the lighter nodes represent the targets participating. The edge represents the interaction between the target and the path, the color of the node is proportional to the degree of interaction. KEGG = Kyoto encyclopedia of genes and genomes.

KEGG pathway enrichment analysis at *P* < .05 significance level was performed for the 91 modified Huanglian Wendan decoction targets. In the bubble diagram, the abscissa represents the *P* value, the color also reflects the enrichment degree, and the bubble size represents the number of genes. The annotation of the KEGG pathway is shown (Figs. 6A and 6B,Table 4, Supplemental Digital Content 8, http://links.lww.com/MD/I643), mainly include: neuroactive ligand receptor interaction, tumor necrosis factor (TNF) signaling pathway, hypoxia-induced factors (HIF)-1 signaling pathway, Calcium signaling pathway, drug metabolism- cytochrome P450.

**Table 4:** Basic information of the top 10 pathways with the most genes.

Term	*P* value	Genes
HSA04080	3.11E–15	CHRM2/OPRD1/GRIA2/CHRM3/PRSS1/CHRM1/CHRNA2/CHRM4/CHRNA7/PTGER3/CHRM5/HTR2C/ADRA1D/ADRB1/ADRB2/HTR2A/ADRA1B/NR3C1/ADRA1A/DRD1/DRD2/DRD3/DRD5/GABRA2/GABRA1/GABRA6/GABRA5/GABRA3/OPRM1/ADRA2C/F2/ADRA2A
HSA04020	3.01E–09	CHRM2/CHRM3/CHRM1/NOS2/NOS3/CHRNA7/PTGER3/CHRM5/HTR2C/ADRA1D/ADRB1/ADRB2/HTR2A/ADRA1B/ADRA1A/EGFR/PPP3CA/DRD1/PRKACA/DRD5
HSA05200	4.29E–09	RB1/GSK3B/GSTP1/PTGER3/PTGS2/EGFR/PIK3CG/RXRB/MAPK8/RXRA/MAPK1/PRKACA/EGLN1/JUN/NOS2/MMP1/EGF/MMP2/VEGFA/AR/IL-6/CDK4/CDK2/BCL2/CYCS/PPARG/MET/TP53/PPARD
HSA04668	2.25E–07	JUN/VCAM1/MMP3/PTGS2/MAPK14/SELE/TNF/PIK3CG/CASP7/IL-6/MAPK8/IL1B/CCL2/MAPK1
HSA04066	4.92E–07	EGLN1/NOS2/NOS3/EGF/INSR/EGFR/PIK3CG/VEGFA/IL-6/IFNG/BCL2/HMOX1/MAPK1
HSA00982	1.00E–06	GSTM2/GSTM1/CYP2B6/MAOB/ADH1C/MAOA/ADH1B/GSTP1/ADH1A/CYP1A2/CYP3A4
HSA05219	1.38E-06	RB1/MMP1/CDK4/EGF/MMP2/MAPK1/TP53/EGFR/VEGFA
HSA05014	6.63E–06	PPP3CA/GRIA2/CAT/BCL2/CYCS/MAPK14/TNF/TP53/SOD1
HSA05142	7.94E–06	IL-6/JUN/MAPK8/IFNG/NOS2/IL1B/MAPK1/CCL2/MAPK14/TNF/IL2/PIK3CG
HSA05222	8.02E–06	RXRB/RB1/RXRA/NOS2/CDK4/CDK2/BCL2/CYCS/PTGS2/TP53/PIK3CG

EGFR = epidermal growth factor receptor, IL-6 = interleukin-6, MAPK8 = mitogen-activated protein kinase 8, TNF = tumor necrosis factor, VEGFA = vascular endothelial growth factor A.

### 3.7. Molecular docking verification

Molecular docking is a technique that mimics the interaction between small ligand molecules and receptor protein macromolecules, and the binding energy between the 2 counterparts can be calculated to predict their affinity. A binding energy lower than 0 indicates that the 2 molecules combine spont- aneously and that smaller binding energies lead to more stable conformations.

**Figure 7.**
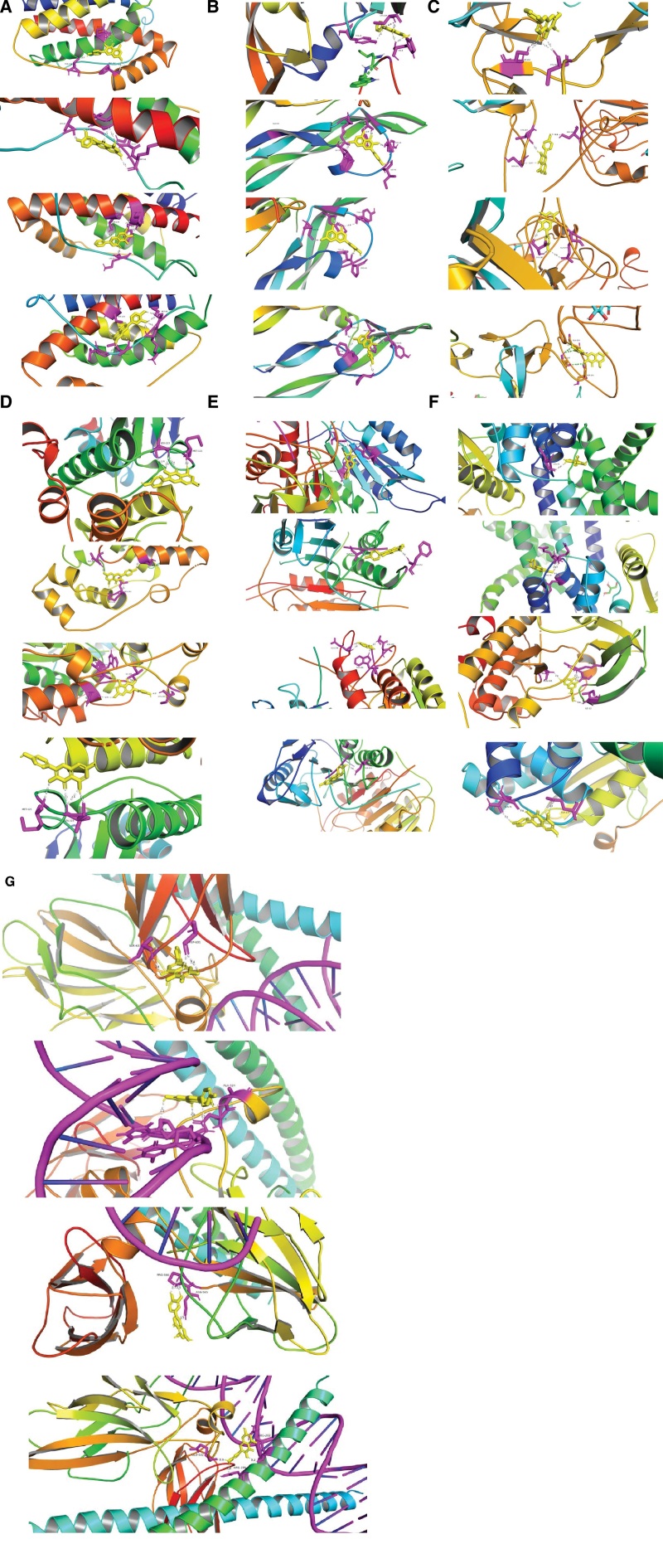
Docking results of target and active ingredient, the details are as follows: (A1–A4) IL-6 and Quercetin, Kaempferol, Baicalei and Luteolin docking results. (7B1–B4) VEGFA and Baicalein, Kaempferol, Luteolin and Quercetin docking results. (C1–C4) EGFR and Baicalein, Kaempferol, Luteolin and Quercetin docking results. (D1–D4) MAPK8 and Baicalein, Kaempferol, Luteolin and Quercetin docking results. (E1–E4) CASP3 and Baicalein, Kaempferol, Luteolin and Quercetin docking results. (F1–F4) CCND1 and Baicalein, Kaempferol, Luteolin and Quercetin docking results. (G1–G4) MAPK8 and Baicalein, Kaempferol, Luteolin and Quercetin docking results. CASP3 = Caspase 3, CCND1 = recombinant cyclin D1, EGFR = epidermal growth factor receptor, IL-6 = interleukin-6, MAPK8 = mitogen-activated protein kinase 8, VEGFA = vascular endothelial growth factor A.

In order to further verify the prediction of the results, and elaborate the mechanism and scientific connotation of modified Huanglian Wendan decoction in the treatment of PCOS, the 4 components with the highest content of modified Huanglian Wendan decoction were selected for molecular docking verification, namely quercetin, kaempferol, luteolin, and baicalein (Table 5). IL-6, VEGFA, EGFR, MAPK8, CCND1, CASP3 and FOS were selected from RCSB protein data, direct docking was performed with grid center 0.066, 0.263,0.166, and NPTS 126 94 108 0.341. The docking results see Figure 7 and Table 6.

**Table 5:** Core components list.

Compound	Degree
Quercetin	77
Kaempferol	34
Luteolin	29
Baicalein	20

**Table 6:** Docking results of active ingredients and key targets (kcal/mol).

Term	Kaempferol (kcal/mol)	Quercetin (kcal/ mol)	Luteolin (kcal/mol)	Baicalein (kcal/mol)
IL-6	−6.94	−7.28	−7.58	−4.5
VEGFA	−8.1	−7.36	−7.84	−6.83
EGFR	−4.66	−4.63	−5.04	−5.92
MAPK8	−4.61	−4.96	−4.87	−4.71
CCND1	−4.59	−4.78	−4.43	−4.84
CASP3	−3.8	−3.6	−3.64	−4.48
FOS	−3.88	−4.07	−3.93	−4.46

CASP3 = Caspase 3, CCND1 = recombinant cyclin D1, EGFR = epidermal growth factor receptor, IL-6 = interleukin-6, MAPK8 = mitogen-activated protein kinase 8, VEGFA = vascular endothelial growth factor A.

## 4. Discussion

Through the 3.4 and 3.5, obtained the active ingredients of modifed Huanglian Wendan decoction for treating PCOS were identified as quercetin, luteolin, kaempferol and baicalein. Modern pharmacological studies showed that quercetin could inhibit TLR/ NF-κB signaling pathway and MAPK signaling pathway, reduce the expression of testosterone (T), estradiol (E2), IL-6 and TNF-α, and increase the expression of FSH, thus alleviating the hormone, metabolism, and ovulation abnormalities caused by PCOS, it can also significantly reduce PCOS-IR and induce expression of GLUT4 and ERa genes in the uterus, therefore effectively treating PCOS and its complications: insulin resistance (IR) and infertility.^[13,14]^ Luteolin was found to regulate the ovarian and hormone levels of rats, up-regulate Nrf2 signaling pathway, and improve antioxidant response, which is of great significance for the prevention and treatment of PCOS.^[15]^ Studies have shown that kaempferol regulated MAPK signaling pathway to reduce inflammatory response and apoptosis, PI3K signaling pathway is mediated to promote meiosis of oocytes.^[16]^ Baicalein regulation of InsR/IRS-1 signaling pathway, inhibits gluconeogenesis of hepatocytes to improve glucose metabolism, has strong anti-IR effect,^[17]^ it restore the dynamic balance of reproductive hormones and normal pregnancy micro-environment through immune regulation, endocrine hormone action network, and improve the antioxidant capacity of mouse ovarian tissues by up-regulating TrxR expression.^[18,19]^ Molecular docking, according to the results of key ingredients and targets combination conformation stability, speculated that these ingredients may be the key ingredients and targets of modified Huanglian Wendan decoction in the treatment of PCOS.

PPI results showed that IL-6, VEGFA, EGFR, MAPK8, CCND1, CASP3, and FOS showed the highest correlation among the core targets. Molecular docking proved that quercetin, luteolin, kaempferol, and baicalin had stable binding to these core targets, suggesting that network pharmacology has a high reliability for mechanism analysis of modified Huanglian Wendan decoction in the treatment of PCOS. IL-6 is a group of cytokines secreted by granulosa cells (GC), Studies have shown that IL-6 plays different roles in follicular generation, ovulation and luteal function, and is involved in the local inflammatory immune response of ovary, which is an influential factor of non-ovulation in PCOS patients.^[13,20]^ Studies have shown that VEGFA is expressed in ovarian GC and membrane cells, as well as in follicular fluid. Abnormal VEGFA expression leads to irregular angiogenesis, which increased ovarian interstitial vascularization and perifollicular angiogenesis, involved in the development and progression of PCOS. In addition, it affects the quality of follicular mature oocytes, during pregnancy and embryo growth and development, high VEGFA concentration in follicular fluid in patients with PCOS can lead to an increase in immature follicles and a decrease in fertilized eggs.^[21–23]^ EGFR is the most abundant expression in human GC and follicular fluid, has been shown to be an ovulation induction factor. When EGFR and P-EGFR are down-regulated, the induction of oocyte maturation is significantly inhibited; EGFR can also delay the meiosis of oocytes by inducing ERK1/ERK2 phosphorylation, thereby regulating ovulation time.^[24–26]^ MAPK8 is a member of the protein mitokine family, which is involved in cell proliferation, cell differentiation, cell apoptosis, and other mechanisms, meanwhile, acts as an integration point for multiple biochemical signals.^[27,28]^ Experiments have confirmed that MAPK8 is closely related to obesity and IR, expression increase in IR in obese mice, higher expression of MAPK increase the start-up process of adipocyte differentiation, promote mature adipocyte differentiation; MAPK8 known as JNK1 gene, involved in initiating the signaling pathway of JNK1/SAPK1 of cell cycle, studies have shown that IR and hyperlipidemia in PCOS patients are related to the high expression of JNK1 mRNA caused by JNK1, speculated that it may be the factor affecting the pathogenesis of PCOS.^[29,30]^ CCND1 regulates cyclinD1 protein and promotes cytoplasmic proliferation, Cyclin D1 and its inhibitors are associated with GC proliferation at specific follicular stages, CCND1 promoted E2 secretion of PCOS ovarian GC, increased the expression level of CYP19A1, inhibited the expression level of Caspase 3 in apoptotic GC, and reduced CASP3 cleavage.^[31,32]^ CASP3 is a regulatory factor of apoptosis signaling pathway, the early ovarian stage of PCOS patients is characterized by increased number of follicles, and the apoptosis rate of sinus follicles is related to increased expression of CASP3 in GC. Hyperandrogenemia in the late stage is closely related to excessive GC apoptosis. GC is the only cell type that can express FSH receptor, and a large number of GC apoptosis occurs. In the process of T conversion to estrogen, androgen will gradually accumulate, and CASP3 will be overexpressed. Similarly, an excess of androgens can lead to an increase in CASP3 and GC, thus leading to a sustained increase in PCOS hyperandrogenemia.^[33–37]^ FOS is nuclear phosphoprotein produced by c-FOS transcription, which plays an important role in regulating cell proliferation, differentiation and apoptosis.^[38]^ The silencing of FOS expression in GC resulted in a significant increase of CYP17 mRNA, suggesting that the FOS family regulates ovarian androgen production.^[39,40]^ FOS can down-regulate the expression of CYP17 in cell membranes and particles, Inhibition of FOS expression in GC resulted in a significant increase in CYP17 expression and androstenedione production, even in this typical non-androstene-derived cell type^[41]^, The abnormal down-regulation of FOS expression in androgen-producing tissues is the potential mechanism of excessive androgen production in PCOS. FOS is the proximal promoter of FSH subunit (FSHB), regulates the expression of FSHB gene. FOS with high expression can induce FSHB (31), FOS expression is low in PCOS patients, while FSH low level is general in PCOS patients, FOS is also involved in p38MAPK/ERK1/2 signal transduction and can be induced by GnRH in gonadotropins, which proves that GnRH regulation of FSHB gene expression may be induced by FOS,^[42,43]^ in addition, some studies have confirmed that the expression of FOS is positively correlated with the expression of inflammatory adipocytokine (IL-6, IL-8) genes, and the mRNA level of the hormone producing factor CYP19A1 is also closely correlated with FOS mRNA (39,40), therefore, it is speculated that the high expression of FOS in PCOS may have an anti-inflammatory effect and have a certain influence on steroid production in adipose tissue, inhibiting the expression of FOS may accelerate the overall development of PCOS, FOS expression was inhibited to accelerate the development of PCOS.^[44–46]^

The functional enrichment results of GO biological process showed that the biological process of modified Huanglian Wendan decoction in treating PCOS mainly focused on oxidative stress, cell proliferation, Regulation of gated ion channels, and other aspects and other aspects. Hypoxia affect human health through regulating system and cell physiology of oxygen can produce reactive oxygen species, cause oxidative stress, and by changing the mammalian ovary oocytes and the physiological function of impact women’s reproductive health, hypoxic conditions induced by a variety of apoptosis signaling pathways, such as autophagy and apoptosis of ovarian follicle mammal necrosis, hypoxia inducing autophagy is through 5’ Amp-activated protein kinase- mammalian target of rapamyciner stress/unfolding protein response and protein kinase C-delta-C-JUNn terminal kinase 1 signaling pathway are performed in a variety of somatic cells,^[47]^ The follicle undergoes many structural changes during growth, including changes in the vasculature cell proliferation and differentiation and formation of fluid-filled cavities, and these changes together create a hypoxic environment within the follicle so that the oocyte itself develops in a potentially hypoxic environment Survival of hypoxic tissues is controlled by HIF, which are activated in hypoxia. Understanding of the HIF signaling pathway is growing in all areas of biology, and its role in ovarian development is steadily being clarified, one of the genes upregulated by HIF is vascular endothelial growth factor, which is a major inducer of angiogenesis and is essential for ovulation and body formation. Ovulation is also intrinsically linked to HIF activity, increasing HIF expression through a surge in luteinizing hormone (LH) through ovulation. The early activation and growth initiation of dormant follicles induced by hypoxia provides evidence that these changes are associated with reduced stromal cell proliferation, suggesting that hypoxia-induced damage to the serst-cell pool may be the mechanism of accelerated follicular activation. The reversal of hypoxia can cause the decrease of SIRT1 activity in mitochondria and during hypoxia.^[48–50]^ The ovarian GC of PCOS patients show abnormal proliferation, which can be reversed by reducing cell proliferation and inhibiting apoptosis with inhibitors.^[51]^ Nitric oxide (NO) plays a role in the physiology of the reproductive system, follicular development and selection, is associated with angiogenic events, play a regulatory role in steroid production in ovarian cells by stimulating cyclooxygenase-2 activity, NO induces increased production of prostaglandin E2, which appears to be a common ovulation mechanism, NO level is negatively correlated with IR in PCOS patients, and the effects of NO and IR are mutually reinforcing, many of the effects of insulin depend on NO, which is an effector in insulin information transduction pathway.^[52,53]^ ERK1 and ERK2 genes are closely related to IR in PCOS. The ERK2-PI3K/AKT and MAPK/ERK pathways in classic insulin-sensitive tissues target ovarian tissue, and disruption of the signaling pathway can lead to decreased insulin sensitivity and compensative hyperinsulinemia in PCOS rats.^[54,55]^ GABA and positive-feedback GABA receptor regulatory steroids, such as isoprogesterone, stimulate food intake and weight gain. In women with PCOS, high serum isoprogeolone concentrations are associated with uncontrolled eating and disturbance of sensitivity to isoprogeolone.^[56]^ Receptor-gated ion channels play an important role in ovarian physiology and pathology. For example, inhibition of volt-gated Ca^2+^ channels can reduce extracellular Ca^2+^ concentration, prevent Ca^2+^ from transmembrane flow, and destroy LH stimulated steroid production in isolated ovarian cells^[57]^, adenylate cyclase- activating polypeptide stimulates melatonin synthesis and melatonin can increase the expression of SIRT1 to inhibit mitochondrial autophagy and protect GC from mitochondrial damage, it can also regulate dehydroepiandrosterone-induced PCOS autophagy through PI3K-Akt pathway, thereby improving ovarian dysfunction and restoring female fertility.^[58–60]^ Studies have shown that GC in patients with PCOS are in an abnormal proliferation state, TNF-α activates JNK and other signal transduction pathways to promote the proliferation of follicular membrane cells, resulting in ovarian white membrane thickening and follicular membrane cell hyperplasia in PCOS patients, and can promote the occurrence of PCOS-IR.^[61–63]^ The binding of acetylcholine (ACH) to the miAChRs receptor in GnRHergic neurons activates the phospholipase C protein, which hydrolyzes phosphatidylinositol and stimulates the formation of inositol triphosphate (IP3). The binding of IP3 to its receptor in the membrane of the rough endoplasmic reticulum moves Ca^2+^ into the cytoplasm, the phosphorylated IP3 receptor decreases its response capacity when IP3 increases, induced by ACH, which results in a reduction in the release of calcium into the cytoplasm by the endoplasmic reticulum and a lower secretion of GnRH.^[64]^ ACH and choline acetyltransferase are present in adult rats, Primates and women mature ovarian follicle in granular cells, can serve as the role of FSH partial mediator role in the growth of follicle, stimulation of GC proliferation and growth of ovarian ACH levels increase, sinus increase our secondary follicle and corpus luteum number, before the ACH to stimulate the manner and ruptured follicle and corpus luteum formation.^[65,66]^ In conclusion, Modified Huanglian Wendan decoction exert antioxidant stress response, regulate gated ion pathway, inhibit abnormal cell proliferation and other ways to achieve IR, regulate steroid hormone disorder, reduce ovarian cell apoptosis, and finally play the role of anti-PCOS.

The KEGG enrichment pathway indicated that the main pathways of midified Huanglian Wendan decoction in treating PCOS were neuroactive ligand receptor interaction, TNF signaling pathway, HIF-1 signaling pathway, Calcium signaling pathway, drug metabolism -cytochrome P450. Neuroactive ligand receptor interaction plays an important role in the reproductive process, studies have confirmed that endometrial gene expression in PCOS patients is related to neuroactive ligand receptor interaction, in addition, neuroactive ligand receptor interaction pathway is related to abnormal ovarian follicular secretion. In this pathway, IL12B and F2R genes participate in the pathophysiological mechanism of PCOS by regulating cell death and cells. Moreover, studies have confirmed that the neuroactive ligand receptor interaction pathway can regulate endocrine and emotion, and emotion regulation has obvious therapeutic effect on PCOS patients.^[67,68]^ TNF signaling pathway is an inflammatory signaling pathway, which is involved in the pathogenesis and development of PCOS. The inflammatory factor IL-6 enriched in this pathway is involved in the local inflammatory immune response of ovary, TNF-α can inhibit FSH synthesis, and both IL-6 and TNF-α can prevent a large number of follicles from growing, Regulation of TNF signaling pathway can play an anti-PCOS role.^[69–73]^ HIF-1α is a multidirectional factor that controls intracellular communication, sterogenic activity and development rate of oocytes, and affects blastocyst rate, inhibition of HIF-1α signaling pathway can not only regulate ovarian cell function, but also reduce inflammatory response and prevent oxidative stress, activation of HIF-1α signaling pathway can stimulate the expression of VEGFA gene, increase endometrial vascular hyperplasia and repair, and provide conditions for implantation of fertilized eggs.^[74,75]^ Calcium signaling pathways involved in oxidative stress reaction, oxidative stress can promote the development of inflammation, induce IR and androgen secretion, studies have shown that calcium disorders can cause ovarian follicles block, cause reproductive and menstrual disorders, resulting in PCOS, when calcium signaling pathways activated, intracellular calcium ion concentration increases, the activation of calmodulin, prompting designed the gonadotropins release, gonadotropins indirectly regulates oocyte maturation through paracrine mechanism, resulting in ovulation, Therefore, for PCOS patients, regulating calcium ion signaling pathway can resist oxidative stress, and then play a role in resisting insulin resistance and inhibiting androgen secretio.^[76–80]^ P450 aromatase is a key enzyme that catalyzes the conversion of androstenedione and T to estrone and E2, increased androgen level in serum of PCOS patients suggests that there may be defective aromatase activity, Sustained high serum levels of LH in PCOS women stimulated the production of large amounts of and rostenedione, in ovarian follicular membrane cells and stromal cells and stimulated the expression of P450 aromatase mRNA, In the study of the effect of insulin sensitizer on PCOS, it was found that metformin can directly act on the ovary, reduce the activity of P450 aromatase, reduce the synthesis of estrogen, and remove the negative feedback of estrogen on FSH in follicular fluid, increase the expression of FSH receptor and promote follicular development.^[81–83]^

In summary, this study adopted network pharmacology and molecular docking methods to identify potential therapeutic targets of modified Huanglian Wendan decoction for PCOS. The relationship between modified Huanglian Wendan decoction and PCOS disease was preliminarily clarified, and the relationship between active molecules and target proteins was visualized, revealing that modified Huanglian Wendan decoction in treating PCOS is a multi-active ingredient that plays an anti-inflammatory and antioxidant stress role through multi-target and multi-pathway However, the prediction is only based on the existing database at present, which can only provide reference for the study of its mechanism of action. The subsequent research group plans to design animal experiments to conduct in-depth discussion on the mechanism of action of modified Huanglian Wendan decoction in the treatment of PCOS, so as to clarify its pharmacological mechanism more systematically and scientifically and provide more reference for its clinical use.

## 5. Conclusion

In summary, this manuscript uses network pharmacology to analyze the effective targets and pathways of adding and modified Huanglian Wendan decoction, and preliminarily explores the target action pathway and molecular mechanism of adding and modified Huanglian Wendan decoction in the treatment of PCOS. This study indicated that the mechanism of modified Huanglian Wendan decoction in treating PCOS was related to its anti-inflammatory, antioxidant stress, regulation of in vivo hormone production and resistance to insulin. The main active components are quercetin, luteolin, kaempferol, and baicalein, and the key targets are IL-6, VEGFA, EGFR, MAPK8, CCND1, CASP3 and FOS. Neuroactive Ligand receptor interaction, TNF Pathway, HIF-1 pathway, Calcium signaling pathway, Drug metabolism- Cytochrome P450 and other signal pathways are correlated, which again confirms that TCM is effective in treating diseases through multiple targets and multiple pathways. Due to many subjective and objective factors, experimental verification needs to be carried out in the next step, which will open up a new way for the treatment of PCOS.

## Acknowledgments

This is work was supported by the fourth batch of National Outstanding Talents of Traditional Chinese Medicine (Clinical and Basic) Research and Training Program of State Administration of Traditional Chinese Medicine (No. 24 [2017] of State Administration of Traditional Chinese Medicine), Shandong Provincial Traditional Chinese Medicine Science and Technology Plan Project (2020M042).

## Author contributions

**Data curation:** Zhaojng Wu, Tiantian Yang.

**Formal analysis:** Zhaojng Wu.

**Funding acquisition:** Hongbo Ma.

**Validation:** Zhaojng Wu.

**Visualization:** Zhaojng Wu.

**Writing – original draft:** Zhaojng Wu.

**Writing – review & editing:** Zhaojng Wu.

## Supplementary Material

















## References

[1] Bazarganipour F Ziaei S Montazeri A. Body image satisfaction and self-esteem status among the patients with polycystic ovary syndrome. Iran J Reprod Med. 2013;11:829–36. 24639704 PMC3941334

[2] Zhang H Deng LQ Xiang SF. Effect of transvaginal ultrasound on the diagnostic accuracy of PCOS. J Appl Obstetrics Gynecol. 2015;31:858–60.

[3] Liu L Li WZ Zou GL. Huanglian wendan tang treatment research progress in metabolic syndrome. Chin J Experimental Formulas Chin Med. 2020;26:190–6.

[4] Ru J Li P Wang J. TCMSP: a database of systems pharmacology for drug discovery from herbal medicines. J Cheminform. 2014;6:13. 24735618 10.1186/1758-2946-6-13 PMC4001360

[5] Davis AP Grondin CJ Johnson RJ. Comparative toxicogenomics database (CTD): update 2021. Nucleic Acids Res. 2021;49:D1138–43. 33068428 10.1093/nar/gkaa891 PMC7779006

[6] Luo JC. Introduction of UniProt protein database. Bioinformatics. 2019;17:131–44.

[7] Doncheva NT Morris JH Gorodkin J. Cytoscape STRING app: network analysis and visualization of proteomics data. J Proteome Res. 2019;18:623–32. 30450911 10.1021/acs.jproteome.8b00702 PMC6800166

[8] Szklarczyk D Gable AL Nastou KC. The STRING database in 2021: customizable protein-protein networks, and functional characterization of user-uploaded gene/measurement sets [published correction appears in Nucleic Acids Res. 2021 Oct 11;49(18):10800]. Nucleic Acids Res. 2021;49:D605–12. 34530444 10.1093/nar/gkab835 PMC8501959

[9] The Gene Ontology Consortium. The gene ontology resource: 20 years and still GOing strong. Nucleic Acids Res. 2019;47:D330–8. 30395331 10.1093/nar/gky1055 PMC6323945

[10] Kanehisa M Goto S. KEGG: kyoto encyclopedia of genes and genomes. Nucleic Acids Res. 2000;28:27–30. 10592173 10.1093/nar/28.1.27 PMC102409

[11] Kim S Chen J Cheng T. PubChem in 2021: new data content and improved web interfaces. Nucleic Acids Res. 2021;49:D1388–95. 33151290 10.1093/nar/gkaa971 PMC7778930

[12] Mooers BHM Brown ME. Templates for writing PyMOL scripts. Protein Sci. 2021;30:262–9. 33179363 10.1002/pro.3997 PMC7737772

[13] Wang Z Zhai D Zhang D. Quercetin decreases insulin resistance in a polycystic ovary syndrome rat model by improving inflammatory microenvironment. Reprod Sci. 2017;24:682–90. 27634381 10.1177/1933719116667218

[14] Neisy A Zal F Seghatoleslam A. Amelioration by quercetin of insulin resistance and uterine GLUT4 and ERα gene expression in rats with polycystic ovary syndrome (PCOS). Reprod Fertil Dev. 2019;31:315–23. 30103849 10.1071/RD18222

[15] Huang Y Zhang X. Luteolin alleviates polycystic ovary syndrome in rats by resolving insulin resistance and oxidative stress. Am J Physiol Endocrinol Metab. 2021;320:E1085–92. 33900851 10.1152/ajpendo.00034.2021

[16] Santos JMS Monte APO Lins TLBG. Kaempferol can be used as the single antioxidant in the in vitro culture medium, stimulating sheep secondary follicle development through the phosphatidylinositol 3-kinase signaling pathway. Theriogenology. 2019;136:86–94. 31254726 10.1016/j.theriogenology.2019.06.036

[17] Yang Z Huang W Zhang J. Baicalein improves glucose metabolism in insulin resistant HepG2 cells. Eur J Pharmacol. 2019;854:187–93. 30970232 10.1016/j.ejphar.2019.04.005

[18] Jiang GJ. Study on Placenta Anticalation and Mechanism of Scutellaria scutellaria and Calamus acorus and their Constituents [D]. Yangzhou University; 2007:20.

[19] Huang Y. Protective effect of baicalin on damage of mouse oocytes induced by triptolide. Chin Herbal Med. 2017;13:4946–51.

[20] Zhu YN Wang Y Wu XK. Expression of interleukin and its receptor in ovarian tissue. J Immunol. 2005;21:75–8.

[21] Tang ZH Yang XF. Expression of VEGF and MIF in the endometrium of patients with polycystic ovary syndrome during window period of implantation. Chin J Maternal Child Health. 2011;26:737–9.

[22] Almawi WY Gammoh E Malalla ZH. Analysis of *VEGFA* variants and changes in VEGF levels underscores the contribution of VEGF to polycystic ovary syndrome. PLoS One. 2016;11:e0165636. 27846231 10.1371/journal.pone.0165636 PMC5112863

[23] Tang ZH Yang XF. Expression of VEGF and MIF in the endometrium of patients with polycystic ovary syndrome during window period of implantation. Chin J Maternal Child Health. 2011;26:737–9.

[24] Wang L. Mechanism of EP300-Mediated EGFR KCR Modification on Oocyte Maturation in Vitro [D]. University of South China; 2019.

[25] Fang L Yu Y Li Y. Upregulation of AREG, EGFR, and HER2 contributes to increased VEGF expression in granulosa cells of patients with OHSS†. Biol Reprod. 2019;101:426–32. 31167229 10.1093/biolre/ioz091

[26] Shimada M Umehara T Hoshino Y. Roles of epidermal growth factor (EGF)-like factor in the ovulation process. Reprod Med Biol. 2016;15:201–216. 29259438 10.1007/s12522-016-0236-x PMC5715866

[27] Kim SW Muise AM Lyons PJ. Regulation of adipogenesis by a transcriptional repressor that modulates MAPK activation. J Biol Chem. 2001;276:10199–206. 11152475 10.1074/jbc.M010640200

[28] Tournier C Hess P Yang DD. Requirement of JNK for stress-induced activation of the cytochrome c-mediated death pathway. Science. 5467;2000:870–4. 10.1126/science.288.5467.870 10797012

[29] Zhong FY QIN ZY LI J. TIAN Hui-qin, HU You-Fang. MIR-484 inhibits the proliferation and differentiation of human precursor adipocytes by targeting MAPK8. J Nanjing Med Univ (Natural Science). 2021;42:325–32.

[30] Osawa H Yamada K Tabara Y. The G/G genotype of a single nucleotide polymorphism at -1066 of c-Jun N-terminal kinase 1 gene (MAPK8) does not affect type 2 diabetes susceptibility despite the specific binding of AP2alpha. Clin Endocrinol (Oxf). 2008;69:36–44. 18036196 10.1111/j.1365-2265.2007.03143.x

[31] Shimizu T Hirai Y Miyamoto A. Expression of cyclins and cyclin-dependent kinase inhibitors in granulosa cells from bovine ovary. Reprod Domest Anim. 2013;48:e65–9. 23631632 10.1111/rda.12177

[32] Zhou X He Y Li N. DNA methylation mediated RSPO2 to promote follicular development in mammals. Cell Death Dis. 2021;12:653. 34175894 10.1038/s41419-021-03941-z PMC8236063

[33] Al Kindi MK Al Essry FS Al Essry FS. Validity of serum testosterone, free androgen index, and calculated free testosterone in women with suspected hyperandrogenism. Oman Med J. 2012;27:471–4. 23226817 10.5001/omj.2012.112 PMC3515039

[34] Barrea L Arnone A Annunziata G. Adherence to the mediterranean diet, dietary patterns and body composition in women with polycystic ovary syndrome (PCOS). Nutrients. 2019;11:2278. 31547562 10.3390/nu11102278 PMC6836220

[35] Chen X Jia X Qiao J. Adipokines in reproductive function: a link between obesity and polycystic ovary syndrome. J Mol Endocrinol. 2013;50:R21–37. 23335807 10.1530/JME-12-0247

[36] Dong ZL Shi-yu H Yan LI. Relationship between free testosterone index and polycystic ovary syndrome with hyperandrogenemia. J Harbin Med Univ. 2012;46:144–6.

[37] Xia XL Jiang HP Ma CS. Male lowering eggs high granulosa cell apoptosis in patients with polycystic ovary syndrome. Influence Guangdong Med. 2015;4:241–4.

[38] Patel SS Beshay VE Escobar JC. Molecular mechanism for repression of 17alpha-hydroxylase expression and androstenedione production in granulosa cells. J Clin Endocrinol Metab. 2009;94:5163–8. 19850690 10.1210/jc.2009-1341

[39] Patel SS Beshay VE Escobar JC. 17α-Hydroxylase (CYP17) expression and subsequent androstenedione production in the human ovary. Reprod Sci. 2010;17:978–86. 20720262 10.1177/1933719110379055

[40] Wang Y Fortin J Lamba P. Activator protein-1 and smad proteins synergistically regulate human follicle-stimulating hormone beta-promoter activity. Endocrinology. 2008;149:5577–91. 18653705 10.1210/en.2008-0220 PMC2584589

[41] Ely HA Mellon PL Coss D. GnRH induces the c-Fos gene via phosphorylation of SRF by the calcium/calmodulin kinase II pathway. Mol Endocrinol. 2011;25:669–80. 21292826 10.1210/me.2010-0437 PMC3063085

[42] Manna PR Dyson MT Stocco DM. Role of basic leucine zipper proteins in transcriptional regulation of the steroidogenic acute regulatory protein gene. Mol Cell Endocrinol. 2009;302:1–11. 19150388 10.1016/j.mce.2008.12.009 PMC5006949

[43] Zhao Y Nichols JE Valdez R. Tumor necrosis factor-alpha stimulates aromatase gene expression in human adipose stromal cells through use of an activating protein-1 binding site upstream of promoter 1.4. Mol Endocrinol. 1996;10:1350–7. 8923461 10.1210/mend.10.11.8923461

[44] Wang L Li H Tang X. Oxidized high-density lipoprotein enhances endocrine disorders and ovarian damage in rats. J Cell Mol Med. 2021;25:8115–26. 34346538 10.1111/jcmm.16197 PMC8419193

[45] Yadav AK Yadav PK Chaudhary GR. Autophagy in hypoxic ovary. Cell Mol Life Sci. 2019;76:3311–22. 31062072 10.1007/s00018-019-03122-4 PMC11105528

[46] Lim M Thompson JG Dunning KR. Hypoxia and reproductive health: hypoxia and ovarian function: follicle development, ovulation, oocyte maturation. Reproduction. 2021;161:F33–40. 33361508 10.1530/REP-20-0509

[47] Gutzeit O Iluz R Ginsberg Y. Perinatal hypoxia leads to primordial follicle activation and premature depletion of ovarian reserve. J Matern Fetal Neonatal Med. 2022;35:7844–8. 34121582 10.1080/14767058.2021.1937985

[48] Nishigaki A Tsubokura H Tsuzuki-Nakao T. Hypoxia: Role of SIRT1 and the protective effect of resveratrol in ovarian function. Reprod Med Biol. 2021;21:e12428. 34934403 10.1002/rmb2.12428 PMC8656197

[49] Bao D Li M Zhou D. MIR-130b-3p is high-expressed in polycystic ovarian syndrome and promotes granulosa cell proliferation by targeting SMAD4. J Steroid Biochem Mol Biol. 2021;209:105844. 33582305 10.1016/j.jsbmb.2021.105844

[50] Basini G Grasselli F. Nitric oxide in follicle development and oocyte competence. Reproduction. 2015;150:R1–9. 25792567 10.1530/REP-14-0524

[51] Nácul AP Andrade CD Schwarz P. Nitric oxide and fibrinogen in polycystic ovary syndrome: associations with insulin resistance and obesity. Eur J Obstet Gynecol Reprod Biol. 2007;133:191–6. 17049715 10.1016/j.ejogrb.2006.09.009

[52] Hu L Zhang Y Chen L. MAPK and ERK polymorphisms are associated with PCOS risk in Chinese women. Oncotarget. 2017;8:100261–100268. 29245975 10.18632/oncotarget.22153 PMC5725017

[53] Xu J Dun J Yang J. Letrozole rat model mimics human polycystic ovarian syndrome and changes in insulin signal pathways. Med Sci Monit. 2020;26:e923073. 32638705 10.12659/MSM.923073 PMC7366789

[54] Holmberg E Sjöstedt J Malinina E. Allopregnanolone involvement in feeding regulation, overeating and obesity. Front Neuroendocrinol. 2018;48:70–7. 28694181 10.1016/j.yfrne.2017.07.002

[55] Paul S Kundu S Pramanick K. Regulation of ovarian steroidogenesis in vitro by gonadotropin in common carp cyprinus carpio: interaction between calcium- and adenylate cyclase-dependent pathways and involvement of ERK signaling cascade. J Mol Endocrinol. 2010;45:207–18. 20668068 10.1677/JME-10-0061

[56] Ma L Li XP Ji HS. Baicalein protects rats with diabetic cardiomyopathy against oxidative stress and inflammation injury via phosphatidylinositol 3-Kinase (PI3K)/AKT pathway. Med Sci Monit. 2018;24:5368–5375. 30070262 10.12659/MSM.911455 PMC6085984

[57] Turhan A Pereira MT Schuler G. Hypoxia-inducible factor (HIF1alpha) inhibition modulates cumulus cell function and affects bovine oocyte maturation in vitro†. Biol Reprod. 2021;104:479–91. 33095229 10.1093/biolre/ioaa196 PMC7876663

[58] Simonneaux V Kienlen-Campard P Loeffler JP. Pharmacological, molecular and functional characterization of vasoactive intestinal polypeptide/pituitary adenylate cyclase-activating polypeptide receptors in the rat pineal gland. Neuroscience. 1998;85:887–96. 9639281 10.1016/s0306-4522(97)00668-4

[59] Yi S Zheng B Zhu Y. Melatonin ameliorates excessive PINK1/Parkin-mediated mitophagy by enhancing SIRT1 expression in granulosa cells of PCOS. Am J Physiol Endocrinol Metab. 2020;319:E91–E101. 32343612 10.1152/ajpendo.00006.2020

[60] Xie F Zhang J Zhai M. Melatonin ameliorates ovarian dysfunction by regulating autophagy in PCOS via the PI3K-Akt pathway. Reproduction. 2021;162:73–82. 33989172 10.1530/REP-20-0643

[61] Hong L Teng XM Li KM. TNF-α promotes the proliferation of porcine follicular intima cells in vitro. West China Med J. 2010;25:46–8.

[62] Korobowicz A. Biology of tumor necrosis factor type alpha (TNF-alpha). Pol Merkur Lekarski. 2006;21:358–61. 17205778

[63] Tarkuni Ceti Narslan B Türemen E. Associa-tion between circulating tumor necrosis factor-alpha, interleukin-6, andinsul in resistance in normal-weight women with polycystic ova-ry syndrome. Metab Syndr Relat Disord. 2006;4:122–8. 18370758 10.1089/met.2006.4.122

[64] Morales A Díaz M Guelmes P. Rapid modulatory effect of estradiol on acetylcholine-induced Ca^2+^ signal is mediated through cyclic-GMP cascade in LHRH-releasing GT1-7 cells. Eur J Neurosci. 2005;22:2207–15. 16262659 10.1111/j.1460-9568.2005.04432.x

[65] Mayerhofer A Kunz L Krieger A. FSH regulates acetycholine production by ovarian granulosa cells. Reprod Biol Endocrinol. 2006;4:37. 16846505 10.1186/1477-7827-4-37 PMC1557511

[66] Urra J Blohberger J Tiszavari M, In vivo blockade of acetylcholinesterase increases intraovarian acetylcholine and enhances follicular development and fertility in the rat. Sci Rep. 2016;6:30129. 27440195 10.1038/srep30129 PMC4954984

[67] Bellver J Martínez-Conejero JA Labarta E. Endometrial gene expression in the window of implantation is altered in obese women especially in association with polycystic ovary syndrome. Fertil Steril. 2011;95:2335–41, 2341.e1. 21481376 10.1016/j.fertnstert.2011.03.021

[68] Liu L He D Wang Y. Integrated analysis of DNA methylation and transcritome profiling of polycystic ovary syndrome. Mol Med Rep. 2020;21:2138–50. 32323770 10.3892/mmr.2020.11005 PMC7115196

[69] Alanbay I Ercan CM Sakinci M. A macrophage activation marker chitotriosidase in women with PCOS: does low-grade chronic inflammation in PCOS relate to PCOS itself or obesity? Arch Gynecol Obstet. 2012;286:1065–71. 22718099 10.1007/s00404-012-2425-0

[70] Oróstica L Astorga I Plaza-Parrochia F. Proinflammatory environment and role of TNF-α in endometrial function of obese women having polycystic ovarian syndrome. Int J Obes (Lond). 2016;40:1715–22. 27569685 10.1038/ijo.2016.154

[71] Ebejer K Calleja-Agius J. The role of cytokines in polycystic ovarian syndrome. Gynecol Endocrinol. 2013;29:536–40. 23368758 10.3109/09513590.2012.760195

[72] Wu HM. The role of TNF-α in the regulation of testosterone secretion and proliferation in follicular cells. Gilling Med J. 2015;36:605–6.

[73] Szczuko M Zapałowska-Chwyć M Drozd A. Changes in the IGF-1 and TNF-α synthesis pathways before and after three-month reduction diet with low glicemic index in women with PCOS. Ginekol Pol. 2018;89:295–303. 30010177 10.5603/GP.a2018.0051

[74] Peitsidis P Agrawal R. Role of vascular endothelial growth factor in women with PCO and PCOS: a systematic review. Reprod Biomed Online. 2010;20:444–52. 20156703 10.1016/j.rbmo.2010.01.007

[75] Artini PG Monti M Cristello F. Vascular endothelial growth factor in females of reproductive age. Gynecol Endocrinol. 2003;17:477–92. 14992167 10.1080/09513590312331290418

[76] Wang F Zhang Z Wang Z. Expression and clinical significance of the HIF-1a/ET-2 signaling pathway during the development and treatment of polycystic ovary syndrome. J Mol Histol. 2015;46:173–81. 25613530 10.1007/s10735-015-9609-4

[77] Cui J Kaandorp JA Ositelu OO. Simulating calcium influx and free calcium concentrations in yeast. Cell Calcium. 2009;45:123–32. 18783827 10.1016/j.ceca.2008.07.005 PMC3130064

[78] Cui J Kaandorp JA. Mathematical modeling of calcium homeostasis in yeast cells. Cell Calcium. 2006;39:337–48. 16445978 10.1016/j.ceca.2005.12.001

[79] Chang JC Lien CF Lee WS. Intermittent hypoxia prevents myocardial mitochondrial Ca^2+^ overload and cell death during ischemia/reperfusion: the role of reactive oxygen species. Cells. 2019;8:564. 31181855 10.3390/cells8060564 PMC6627395

[80] González F Rote NS Minium J. Reactive oxygen species-induced oxidative stress in the development of insulin resistance and hyperandrogenism in polycystic ovary syndrome. J Clin Endocrinol Metab. 2006;91:336–40. 16249279 10.1210/jc.2005-1696

[81] Ashidi B Haghollahi F Shariat M. The effects of calcium-vitamin D and metformin on polycystic ovary syndrome: a pilot study. Taiwan J Obstet Gynecol. 2009;48:142–7. 19574176 10.1016/S1028-4559(09)60275-8

[82] Auvray P Nativelle C Bureau R. Study of substrate specificity of human aromatase by site directed mutagenesis. Eur J Biochem. 2002;269:1393–405. 11874453 10.1046/j.1432-1033.2002.02779.x

[83] la Marca A Morgante G Palumbo M. Insulin-lowering treatment reduces aromatase activity in response to follicle-stimulating hormone in women with polycystic ovary syndrome. Fertil Steril. 2002;78:1234–9. 12477517 10.1016/s0015-0282(02)04346-7

